# HIV-1 Infection and Glucose Metabolism Reprogramming of T Cells: Another Approach Toward Functional Cure and Reservoir Eradication

**DOI:** 10.3389/fimmu.2020.572677

**Published:** 2020-10-07

**Authors:** Shuang Kang, Hong Tang

**Affiliations:** ^1^Division of Infectious Diseases, State Key Laboratory of Biotherapy and Center of Infectious Diseases, West China Hospital, Sichuan University, Chengdu, China; ^2^Center of Infectious Diseases, West China Hospital of Sichuan University, Chengdu, China

**Keywords:** immune reprogramming, immunometabolism, chronic inflammation, HIV reservoir, HIV/AIDS

## Abstract

With the emerging of highly active antiretroviral therapy, HIV-1 infection has transferred from a fatal threat to a chronic disease that could be managed. Nevertheless, inextricable systemic immune activation and chronic inflammation despite viral suppression render patients still at higher risk of HIV-1-associated non-AIDS complications. Immunometabolism has nowadays raised more and more attention for that targeting metabolism may become a promising approach to modulate immune system and play a role in treating cancer, HIV-1 infection and autoimmune diseases. HIV-1 mainly infects CD4+ T cells and accumulating evidence has brought to light the association between T cell metabolism reprogramming and HIV-1 pathogenesis. Here, we will focus on the interplay of glycometabolism reprogramming of T cells and HIV-1 infection, making an effort to delineate the possibility of utilizing immunometabolism as a new target towards HIV-1 management and even sterilizing cure through eliminating viral reservoir.

## Introduction

Highly active anti-retroviral therapy (HAART) has changed the death sentence of human immunodeficiency virus 1(HIV-1) infection into a manageable state comparable to certain chronic diseases such as hypertension and diabetes mellitus. Despite the success that most patients could achieve viral suppression and optimal immune reconstitution with HAART, HIV-1–infected patients remain at higher risk for complications and displayed aging signs at an earlier stage than HIV-1 negative population (1, 2), for which persistent immune activation and chronic inflammation have long been blamed. This may probably result from the complicated interaction of different factors including virus reservoir, intestinal bacterial translocation, and coinfection or reactivation of other pathogens (3). The underlying mechanism is yet to be elucidated.

As one basic feature of cells, energy metabolism not only fuels all kinds of biological activities but also participate in the signal cascades and regulate cellular functions. Technologic development has allowed quantification of cellular metabolic level and brought more and more attention to researches on cell metabolism and immune response, especially in the field of oncology and autoimmune diseases. HIV-1 infection perturbs the immune system and shares similar characteristics with certain autoimmune diseases such as inflammatory bowel disease. Therefore, the interaction between immune cell metabolism and HIV-1 pathogenesis deserves deep exploration and may lead to new therapeutic strategies against HIV-1.

## Mini Review

### Glucose Metabolism and T Cell Function

Glucose is one of the most important sources of energy supply. Cells utilize glucose through oxidative phosphorylation (OXPHOS) and glycolysis to produce adenosine triphosphate (ATP) as a direct energy source. Generally, T cells intake glucose through glucose transporter 1(Glut1) on the membrane and after phosphorylation by hexokinase (HK), one molecule of glucose is converted to two molecules of pyruvate. Then the metabolic pathway bifurcates to meet the needs of different cell functions. Resting differentiated T cells primarily metabolize glucose *via* mitochondrial tricarboxylic acid (TCA) cycle and produce nicotinamide adenine dinucleotide to fuel OXPHOS. Only under anaerobic conditions, glycolysis is upregulated and produces large amounts of lactate with less ATP. Most cancer cells produce large amounts of lactate regardless of the availability of oxygen and this “aerobic glycolysis” was first observed by Otto Warburg and thus known as Warburg effect (4). Despite the massive energy demand to proliferate and function, proliferating mammalian cells including activated T cells significantly upregulate the relatively inefficient aerobic glycolysis, converting pyruvate into lactate even with enough oxygen, the process of which demands no participation of mitochondria but produces less ATP (5–7). Nevertheless, faster ATP generation through aerobic glycolysis can also ensure the energy supply despite the inefficiency (at least in free ATP production). Another important explanation is that production of daughter cells through mitosis require the synthesis of all the cellular component rapidly to mount immune response soon after stimulation. With the intermediates of aerobic glycolysis serving as biosynthesis precursors as well as fast ATP production, the process could be substantially accelerated to generate new cells and produce functional substances (8, 9).

**Figure 1:**
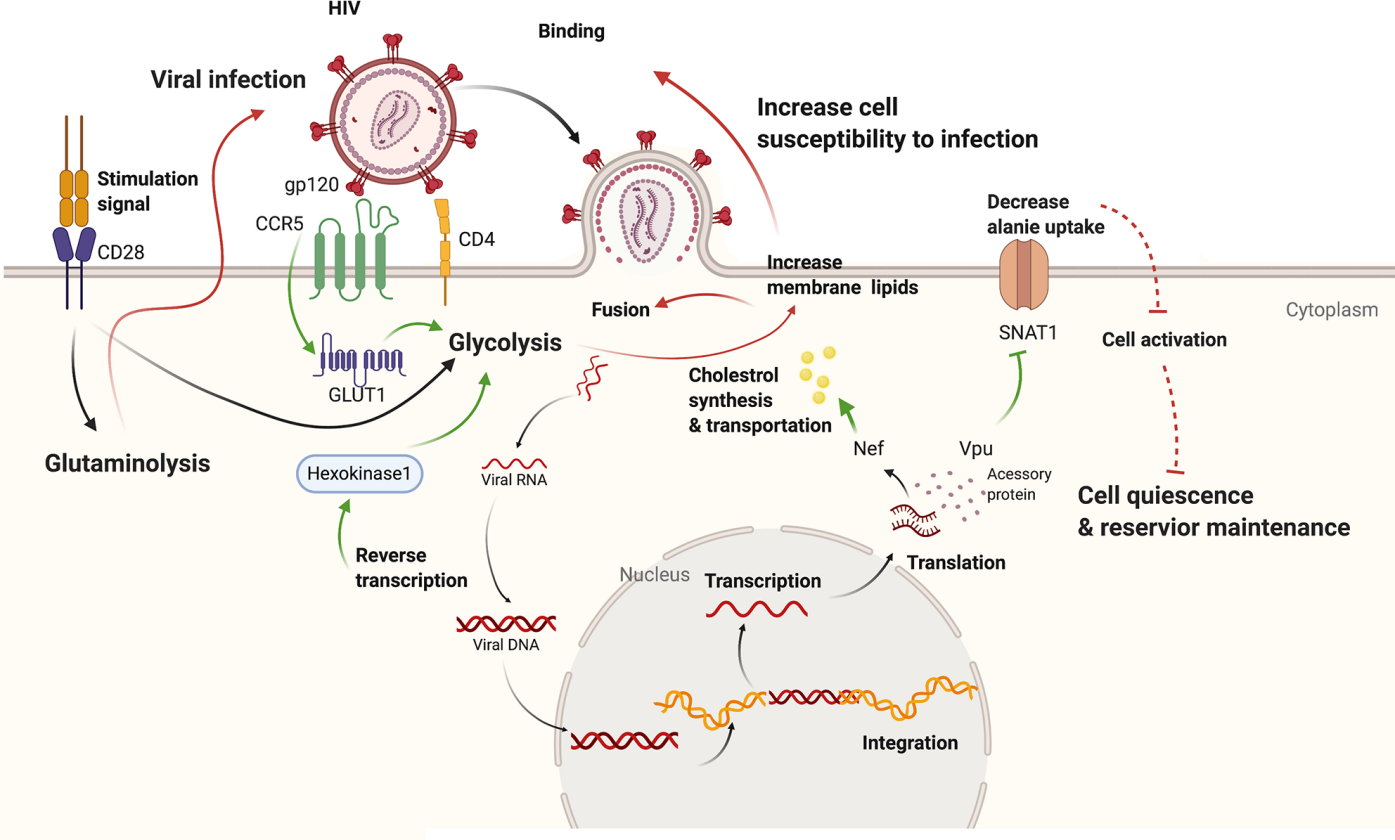
The interplay between HIV infection and host metabolism. HIV infection induces increased glycolysis through promoting Glut1 and HK1. Besides, viral accessory protein Vpu could dampen alanine uptake and Nef could promote cholesterol synthesis and transport. On the other hand, increased glycolysis of host cells leads to increased permission of viral infection as well as viral amplification from reservoir. While downregulated alanine metabolism might promote quiescence in host cells and reservoir maintenance. Effect of HIV-1 infection on cell metabolism is shown with green arrows while effect of altered cell metabolism on HIV-1 pathogenesis with red marks. CCR5, chemokine C-C motif receptor 5; CD4, cluster of differentiation 4; CD28, cluster of differentiation 28; Glut1, Glucose transporter 1; gp120, glycoprotein 120; Nef, negative regulatory factor; SNAT1, serotonin N-acetyltransferase 1; Vpu, Viral protein U.

Metabolic reprogramming plays pivotal role in T cell activation, differentiation and colony expansion. After activation, naïve or memory T cells reinforce a metabolic program conducive to aerobic glycolysis and effector differentiation through PI3K/Akt/mTOR signaling pathway (as shown in **Figure 1**), which has long been recognized as a classic pathway promoting glucose metabolism. Co-stimulation signal of CD28 activates phosphatidylinositol 3-kinase (PI3K) and generates phosphatidylinositol-3-phosphate, further promoting protein kinase B (PKB/Akt) recruitment and activation. Next, the mammalian target of rapamycin complex (mTORC) signaling is turned on. Akt facilitates the transfer of Glut1 to the cell membrane (10) and HK to mitochondria as well as enhance the activity of the latter (11). mTORC1 acts post-transcriptionally through phosphorylating 70KDa ribosomal protein S6 kinase 1(p70S6K1). Studies found that inhibition of p70S6K1 suppressed glycolysis and induced apoptosis in hematopoietic progenitor cells (12). Both CD4+ and CD8+ T cells display enhanced glycolysis after activation while CD8+ T cells are found to be more glycolytic and better able to utilize glutamate, correlating to their increased capacity for growth and proliferation (5, 13). Although glycolysis plays indispensable role in T cell activation, OXPHOS is also upregulated after T cell activation (5, 14). Co-stimulation can contribute to enhanced mitochondria respiratory functions after activation (15) and mitochondria reactive oxygen species (mROS) is required for activation of nuclear factor of activated T cells (NFAT) and subsequent IL-2 induction (16). Glutaminolysis is another important metabolic pathway significantly elevated after T cell activation, supported by the fact that treatment with glutamine antagonist suppresses T cell proliferation (17, 18). Glutamine is major carbon source of a-ketoglutarate (a-KG), an anapleurotic substrate of the TCA cycle (19). Carbon tracing studies demonstrated the incorporation of glutamine carbons into intermediate metabolites in the TCA cycle (20), which could be interpreted as that glutamine fuels mitochondrial ATP production in activated T cells. With multiple energy-generating pathway elevated in T cell activation, it’s hard to determine which is the most indispensable at present, partially since the pathways are closely interconnected.

Different T cell subsets exhibit unique metabolic profiles. As mentioned above, resting T cells undergo dramatic metabolic reprogramming after activation to fulfill corresponding function. With relatively low metabolic demands, T_N_ cells mainly generate energy *via* OXPHOS (21) and have the preference for fatty acid oxidation(FAO). At the same time, T_N_ cells are equipped with a poised ribosomal machinery and pre-accumulation of mRNAs encoding for enzymes involved in glycolysis and fatty acid synthesis (22), to quickly switch to the activated mode upon stimulation. Effector T cells display robust aerobic glycolysis to maximize macromolecule synthesis and energy generation. After the immune response subsides, the effector T cell population contracts and differentiates into long-lived memory cells with key metabolic pathway associated with effector function downregulated, such as glycolysis and mTOR activity (23). Although memory T cells generally exhibit more dependency on OXPHOS compared with effector cells, this population can be further classified as central memory (T_CM_), effector memory (T_EM_), tissue resident memory (T_RM_), virtual memory and stem-cell memory subsets (24), all displaying unique metabolic profiles. T_CM_ cells reside in secondary lymphoid tissues screening for re-appearance of Ag and are the important reservoir containing replication-competent HIV-1 DNA (25). T_CM_ cells are relatively quiescent, making it difficult to recognize these viral seeds and is reported as the major to HIV-1 reservoirs in several studies (25, 26). T_CM_ cells from both mice and humans have more dominant mitochondrial metabolic profiles compared with T_EM_ cells and augmenting aerobic glycolysis negatively impacts T_CM_ cell formation (27, 28), indicating the preference of T_CM_ cells on OXPHOS. T_EM_ cells patrol in peripheral tissues and differentiate more rapidly into effector cells on antigen activation (29, 30). Peripheral tissues are barren in oxygen and nutrition under pathological conditions such as tumors and infection (31, 32), where the T_EM_ cells have to play the role (33, 34). T_EM_ cells are found to have greater spare respiratory capacity and more abundant cytosolic GAPDH as a surrogate of glycolysis reserve (35), allowing for rapid adaptation to the asperity and effective function. Further study found that T_EM_ cells rely less on lipid metabolism under glucose-limiting conditions than T_N_ and T_CM_ cells (36). Besides, IFNg-production could be regulated through changing the lipid levels in glucose-limiting culture media, indicating that relative ratio of glycolysis to fatty acid metabolism within a single effector T cell determines its functional capabilities (36). In the context of HIV-1 infection, TEM cells are found to contain the largest proportion of genetically intact HIV-1 proviruses among memory cell subsets using Full-Length Individual Proviral Sequencing assay (37) while T^CM^ cells are proved as such with QVOA (25). Distinct assays and patient heterogeneity in these studies may contribute to the discrepancies. But it’s commonly recognized that replication-competent proviruses are distributed unequally between the memory CD4+ subsets. The influence of cellular metabolic profiles on the preference of reservoir establishment and maintenance, if any, needs more exploration.

In turn, metabolic milieu orchestrates the differentiation and function of T cells. Effector CD4+ T cells including Th1, Th2, and Th17 mainly rely on aerobic glycolysis to fuel immune response while the anti-inflammatory regulatory T (Treg) cells oxidize fatty acids as an energy source (38, 39). Enzymes of glucose metabolism work pluralistically to regulate immune cell functions. One key enzyme of glycolysis, glyceraldehyde triphosphate dehydrogenase (GAPDH), could bind to the untranslated elements of IFNg mRNA and dampen IFNg production (40). Thus, active glycolysis with the engagement of GAPDH is essential for effector cytokine production including IL-2 and IFNg (40). Whilst lactate dehydrogenase A (LDHA) acts epigenetically by increasing concentrations of acetyl-coenzyme A to enhance histone acetylation and transcription of Ifng (41). Besides, glucose-deprivation also inhibits INFg, GM-CSF and TNFa production in CD8+ T cells, completely or partially (42, 43). Moreover, metabolic regulation could orient T cell differentiation. Researchers found that inhibition of glycolysis suppressed Th1 differentiation and IFN-γ production both translationally and transcriptionally (44). The importance of glucose metabolism is further substantiated by the finding that ablation of mTOR signaling prevents naive CD4+ T cells from differentiating into pro-inflammatory effector cells while selective activation of mTORC1 or mTORC2 renders distinct differentiation into Th1 and Th17 or Th2 (45, 46). Since CD4+ T cells expressing chemokine receptor CCR6, a marker for Th17 lineage polarization, are found to be more permissive to HIV-1 infection (47, 48), the cellular metabolism could be hijacked by the virus to promote infection-favoring differentiation, i.e. promoting CD4+ T cell differentiation towards more virus-susceptible lineage such as Th17.

### The Interplay Between T-Cell Metabolism and HIV-1 Pathogenesis

#### HIV-1 Infection Changes Cellular Metabolism of CD4+ T Cells

Accumulating evidence demonstrates that HIV-1 infection induces an increment of aerobic glycolysis in CD4+ T cells. Early in 1996, Sorbara et al. (49) observed increased expression of GLUT3 and GLUT1 RNA and protein in H9 lymphocytic cells, accompanied by an increase in glucose transport in the infected cells. Glut contains a family of glucose transporters and T cells mainly express Glut1 while Glut 3 is principally found on neurons. The intensified glycolysis induced by HIV-1 infection is further authenticated by Palmer et al. (50) through the analysis of peripheral blood mononuclear cells of HIV-1–infected patients. Measurements of glycolysis markers (Glut1 expression, glucose uptake, intracellular glucose-6-phosphate, and L-lactate) revealed up-regulated glycolysis in CD4+ T cells from HIV-1+ patients, which was not fully revised by ART (50). This might result from the interaction of viral protein gp120 with membrane co-receptor C-C motif receptor 5 (CCR5), since activation of CCR5 by its ligand C-C motif ligand 5 has been shown to promote surface expression of Glut1 and glycolysis in breast cancer cell lines (51). Moreover, the proportion of CD4+Glut1+ cells correlated positively with immune activation defined by expression of HLA-DR and CD38 (50, 52) and inversely with the absolute CD4+ cell counts (50). Enhanced hexokinase activity in HIV-1–infected CD4+ T cells also contributes to the metabolism reprogramming process and this remodeling is shown to depend on viral reverse transcription rather than accessory viral proteins (53).

Downregulation of alanine uptake is another impact of HIV-1 infection on cellular metabolism. A range of transmembrane transporters without well-characterized roles in the immune system were shown to be downregulated by HIV-1 infection through cell surface proteomic map. Among these, the amino acid transporter serotonin N-acetyltransferase 1(SNAT1) is found to be target for endolysosomal degradation by accessory viral protein Vpu (54). Although known as an neuronal glutamine transporter, SNAT1is found to mediate quantitatively important alanine transport in primary CD4+ T cells through consumption and release metabolomics (54). Whilst alanine is a non-essential amino acid and excreted by proliferating cancer cells (55), it was shown to be required for optimal mitogenesis in primary human CD4+ T cells (54). The importance of alanine for T cell activation is further substantiated by the finding that access to extracellular alanine is essential during early T cell activation and memory T cell re-stimulation to fuel protein synthesis (56). T cell activation facilitates viral replication but triggers cell death, which limiting the life-span of infected cells. It’s reasonable to speculate that downregulation of alanine uptake by Vpu facilitates seeding and maintenance of viral reservoir through promoting T cell quiescence.

#### Higher Metabolic Activity of CD4+ T Cells Promotes HIV-1 Infection, Reservoir Replication, and Inflammation

Metabolically highly active CD4+ T cells seems more prone to HIV-1 infection. The consensus is that HIV-1 preferentially infects activated CD4+ T cells, leaving the quiescent subsets unaffected. Immunometabolism studies suggest that metabolically active cells are more prone to HIV-1 infection. CD4+ T cells expressing Glut1 show higher levels of activation marker as well as HIV-1 co-receptor CCR5 compared with CD4+Glut1- cells and blockade of glycolysis pathway by PI3K inhibitor could suppress HIV-1 infection *in vitro* (52). A former study has found IL-7 stimulation could render quiescent T cells permissive to HIV-1 infection (57). Reseaches advancedly disclosed upregulated Glut1 expression as the underlying mechanism and verified the association by that siRNA-mediated Glut1 down-regulation could abrogate HIV-1 infection of quiescent CD4+ T cells (53, 58). These findings lead to the pertinent hypothesis that cellular metabolic activity parallels immune phenotype and both could reflect susceptibility to HIV-1 infection. However, a recent study observed a parallel association of HIV-1 susceptibility with metabolic activity but not with HLA-DR expression (59). In this study, it turned out that only highly glycolytic T_N_ and T_CM_ were observed to express the GFP as a marker of HIV infection after activation, while weakly glycolytic cells were strongly resistant to infection (59). This Discrepancies might be attributed to differences in study designs and the specific mechanism under the sweet tooth of HIV-1 infection remains ambiguous. But the association between T cell glycolysis activity and susceptibility to HIV infection is consistently demonstrated in multiple independent studies and is verified by the impaired viral infection due to inhibition of glycolysis (52, 59, 60). An elegantly designed study utilizing single virus tracking delved into the underlying mechanism and showed that glycolysis activity impacts HIV-1 infection at the fusion stage through altering the T cell membrane order and tension (61). Membrane order and tension are significant contributors to several membrane-regulated processes in human cells important for viral infection, including viral entry and egress (62, 63). Active glycolysis is needed to maitain the membrane status favoring HIV-1 fusion, i.e. higher lipid order and lower levels of membrane tension (61). This study provides in-depth understanding of the importance of cellular metabolism in HIV-1 infeciton and inspires future related researches.

As glutamine catabolism is another metabolic pathway closely associated with T cell activation and function, it’s no wonder that glutamine metabolism is demonstrated with some impact on HIV infection. One study analyzed the effect of different metabolic pathway blockade on HIV infection with metabolic inhibitors and found that inhibition of glycolysis as well as glutaminolysis could both reduce HIV infection, significantly or to heterogeneous extent, respectively (59). Things come out differently in another study by Clerc et al., where they found glutaminolysis as the major pathway fueling OXPHOS in activated T_N_ and T_CM_, and HIV infection of CD4+ T cells was more impacted by deprivation of glutamine than of glucose, with a mean reduction of 73–77% in the former, compared with only a 15–29% reduction in the latter (64). They also found that under attenuated glycolysis, infection was increased with glucose shunted into TCA cycle (64). It should be noted that different metrics of HIV infection were adopted across studies, that is, protein expression of GFP inserted into viral genome for the study by Valle-Casuso et al. (59) and Palmer et al. (52) and mRNA production of reverse transcription for the study by Clerc et al. (64). Thus, the infection was measured at different stage and this could contribute to the controversy of results. Besides, the possibility could not be excluded that there is a priority rank of metabolic pathways fueling infection and that the virus could wire cellular metabolism towards the second preferred one when the first choice is not available.

Cellular metabolism is also a major determinant of HIV-1 replication and latency. By providing CD4+ T cells with a poor substrate for glycolysis, namely galactose, free virions in the culture supernatant was reduced to between 20% and 60% of the amount produced in cultures containing glucose (60). Valle-Casuso et al. (59) found consistent observations and further demonstrated that 2-deoxyglucose-mediated suboptimal inhibition of glycolysis induces the death of infected CD4+ T cells and impairs HIV-1 amplification from CD4+ T cell reservoirs. Therefore, the need of HIV-1 replication and latency for highly glycolytic cells reveals a vulnerability that can be exploited to tackle infection. That inhibition of mTOR activity potently suppresses viremia as well as reversal of latency in mice models and patient cells further substantiates the impact of metabolic remodeling in HIV-1 infection (65, 66). Besides, mTOR administration exhibits ideal immune modulation effect by decreasing the cytokine-associated toxicity induced by T cell activation but not impairing the impair cytotoxic T lymphocyte (CTL) recognition and killing of infected cells (67).

Hypoxia inducible factor 1a (HIF-1a) is one of the most studied immunometabolic regulatory factors in HIV-1 infection. HIF-1 is a heterodimeric transcriptional factor that plays a central role in coordinating cellular energy metabolism and function. Degradation of its subunit HIF-1a is reduced under hypoxia condition and activation of HIF-1a signaling promotes glycolysis through Glut1 and numerous glycolytic enzymes (68, 69). HIV-1 dsDNA induces mitochondrial reactive oxygen species (mtROS) production in infected CD4+ T cells and further upregulates inflammatory cytokine secretion including IL-6, IL-1b through HIF-1a signaling pathway (70). Also, extracellular vesicles from infected CD4+ T cells impetus increased expression of HIF-1a in bystander cells, exacerbating systemic inflammation (70). HIF-1a was shown to induce expression of HIV-1 co-receptor CXCR4 on CD4+ T cells and promote virus infection (71). HIF-1 also participates in the induction of Th17/Treg differentiation (68), the imbalance of which is found to be related to chronic inflammation and suboptimal immune reconstitution in HIV-1 patients (72). HIF-1 activates the signature transcription factor of Th17, retinoic acid-related orphan receptor γt (RORγt), and primes naïve CD4+ T cells toward Th17 differentiation (73). Moreover, heightened HIF-1 level promotes the longevity of Th17 cells through controlling Notch signaling and antiapoptotic gene expression (74) as well as negatively modifies Treg function into an abhorrent Th1 effector-like phenotype (75, 76), profoundly boosting the inflammatory process.

#### Metabolic Plasticity of CD8+ T Cells Is Associated With Viral Control

HIV-1 controllers (HICs) are a rare subset of individuals with an exquisite natural ability to control virus replication in the absence of any therapy and CD8+ T cells play indispensable role in HIV-1 control and possible elimination. Researchers have focused on the unique CD8+ characteristics of this providential population in an effort to elucidate targets of therapeutic interventions. Present evidences make an emphasis on CD8+ T cell features including memory phenotype status (77–79), polyfunctionality (80, 81), proliferative capacity (82, 83), and pro-survival phenotype (84). Considering the intricate association between metabolism with cellular function and longevity, studies begin looking into the metabolic profile of HIC. mRNA microarray analysis demonstrated that CD8+ T cells from HICs exhibited activation of closely related metabolic pathways centered around the eukaryotic initiation factor 2 (eIF2), the mTOR and PI3K (85). Transcripts involved in oxidative stress and hypoxia were also increased in HICs from this study. Another group found contradictory features of HICs compared with HAART treated subjects, that CD8+ T cells from HICs show lower levels of HIF-1A gene expression and higher of VHL, a counteracting gene of HIF-1 (86), implying that cells from HICs are less primed for glycolysis and activation. The link between metabolism profile and HIV-1 control is further verified by the longitudinal analysis of transient HICs which observed deregulation of metabolites mainly involved in glycolysis, TCA and amino acid metabolism associated with changed Gag-specific CD8+ T cells response preceding loss of viral control (87). After metabolic reprogramming through IL-15 treatment, CD8+ T cells from non-controllers displayed increased fatty acid uptake and mitochondrial respiratory capacity together with enhanced HIV-1-suppressing capacity (86). Discrepancy of these findings might be caused by the intrinsic heterogeneity of HIC population (88) but the prospect of metabolic plasticity as a promising new therapeutic strategy toward HIV-1 control is undeniable.

### Potential Therapeutic Approaches Targeting Cellular Metabolism and Challenges

Growing understanding of the immunometabolic changes in HIV-1 infection has raised the wonder whether cellular metabolism can be targeted as a new therapeutic approach towards cure. Since it’s quite an emerging field still to be explored, attempts to test those speculations except for *in vitro* experiments are yet extremely scarce. But it won’t harm to propose such promising strategies. Various studies have shown the dependency of HIV-1 infection and replication on glycolysis and glutaminolysis, and inhibition of these pathways with galactose, 2-DG or 6-diazo-5-oxo-l-norleucine could substantively repress viral infection (58, 59, 64). Survival and anti-viral function of CD8+ T cell is also found to be regulated by metabolic pathways (27, 42, 43, 89). Rescinding the glycolysis dependency and restoring mitochondrial function might help enhance the anti-viral function of CD8+ T cells. Enhanced CD8+ T cell function combined with “shock and kill” strategy might contribute to reservoir shrinkage and remission-free treatment pause. A pilot study assessing the effect of metformin on HIV reservoir in non-diabetic ART-treated individuals is already underway (90). Metformin is an upstream inhibitor of mTOR, functioning through activation of AMPK, thus modulating glycolysis in T cells. Moreover, metformin is proved to normalize mitochondrial dysfunction of CD4+ T cells and alleviate aging-related inflammation (91). Cohort study of PLWH with diabetes revealed the association of metformin treatment with improved CD4+ T cell recovery (92). Thus, the attempt of metformin treatment in non-diabetic HIV-1–infected patients is based on the hypothesis that through normalizing mitochondrial function and regulating glycolysis, metformin might provide anti-inflammatory effect and help restore CD8+ T cell function (93). Besides, inhibiting glycolysis to inhibit further reservoir activation and replication provides another option to fulfil the block and lock strategy. But massive challenges remain when it comes to clinical settings. Whether the unique phenotypes observed ex vivo could represent the situations under physical conditions still needs to be determined. Moreover, cellular metabolism is critical and quite “individualized”, so precise intervention to target cell subsets is warranted so as to keep other regular cell functions undisturbed. Drug delivering system in oncology might provide some clues, such as active-ingredient-loaded nanoparticle directed by specific cell recognizing ligand coating. A recent study has displayed the formation of latency reverse agent-loaded anti-CD4 liposomes which could be selectively uptaken by CD4+ T cells (94). Then comes the question how to recognize virus-bearing CD4+ T cells during chronic infection, considering their quiescent status, their extremely scarce frequency in peripheral blood and their hard-to-access location in the tissue. CD4+ T cells expressing CD32a has been reported as rich in inducible replication-competent proviruses and CD32a might facilitate the reservoir targeting (95). It’s also challenging to develop the regimen to satisfy unique metabolic preferences of different cell subsets to optimize their function towards viral eradication. It’s quite difficult to tackle so much problems with one single strategy, thus combined treatment assisted with metabolic regulators might be the answer.

### Discussion

In light of the link between glucose metabolism and immune function, more and more work concentrates on immunometabolism during HIV-1 infection. The unneglectable pivotal role of glucose metabolism mediated by Glut1, mTOR, and HIF-1 in HIV-1 replication, latency and inflammatory responses has come to light and inspires new therapeutic strategies. Briefly, CD4+ T cells increase glycolysis activity in response to HIV-1 infection and this metabolic reprogramming reciprocally facilitates virus invasion, latency formation, and inflammation. Moreover, glucose metabolism of CD8+ T cells correlates closely with viral suppression capacity.

The current profiling of glucose metabolism of CD4+ T cells in HIV-1–infected patients provide us with the basic outlines of the association between cellular metabolism and HIV-1 infection pathology. Besides, there is accumulating understanding of the importance of amino acid metabolism in HIV-1 pathogenesis, such glutamine and alanine. However, more comprehensive observations are needed concerning different metabolic pathways including glucose, lipid and amino acid metabolism and the metabolic profiles in HIV-1–infected patients with different clinical characteristics such as those with optimal immune recovery or not, so as to further utilize cellular metabolism as a treatment target toward better outcomes. On the other hand, oncologists have gained some experience of taking metabolic features of CD8+ T cells, such as glycolysis- and glutaminolysis-targeted approaches, as a therapeutic target to enhance antitumor efficacy. While hyperactivation of glycolysis seems like an unwanted feature of immune cells during HIV-1 infection, the antitumor functions of CD8+ cells in the field of oncology display preference and necessity of glucoses as energy source. Certain researches with elaborated experiments have supplied rigid evidence that reduced glycolysis in CD8+ effector cells inhibits their antitumor functions (96). The inhibition marker PD-1 was also reported to dampen antitumor immune response through inhibiting glycolysis and promoting FAO in T cells (97). The dis-accordance might be attributed to the different process of eliciting immune response in tumor and chronic infection——It took long time for immune system to mount response to tumors while the pathogenetic signal often surges and gradually simmers down in chronic infection. Furthermore, researches concerning tumor immune response usually adopt mice model and cell culture *ex vivo*, which can be very different from primary human cells *in vivo*. Anyhow, more explorations are warranted to figure out the cellular metabolic changes of CD8+ cells as well as its association with anti-viral function during HIV-1 infection.

Metabolic regulating strategies such as metformin administration have gained some success in intensifying cancer immunotherapy. Future studies are required to evaluate the efficacy and safety of these agents in HIV-1 patients as well as to elucidate the impact of other metabolic processes including mitochondrial biogenesis and amino acid metabolism.

## Author Contributions

SK drafted the manuscript and HT provided guidance and fund support. All authors contributed to the article and approved the submitted version.

## Funding

This work was supported by the 1.3.5 Project for Disciplines of Excellence, West China Hospital, Sichuan University (No. ZYGD20009), Sichuan Science and Technology Program (2019JDR0354) and Fund for Nursing Development of West China Hospital (HXHL19035).

## Conflict of Interest

The authors declare that the research was conducted in the absence of any commercial or financial relationships that could be construed as a potential conflict of interest.
